# Arterial stiffness and kidney disease progression in the systolic blood pressure intervention trial 

**DOI:** 10.5414/CN109982

**Published:** 2020-05-25

**Authors:** Kristen L. Nowak, Michel Chonchol, Anna Jovanovich, Zhiying You, Walter T. Ambrosius, Monique E. Cho, Stephen Glasser, James Lash, Debra L. Simmons, Addison Taylor, Daniel Weiner, Anjay Rastogi, Suzanne Oparil, Mark A. Supiano

**Affiliations:** 1 Division of Renal Diseases and Hypertension, University of Colorado Anschutz Medical Campus,; 2 Rocky Mountain Regional VA, Aurora, CO,; 3 Department of Biostatistics and Data Sciences, Division of Public Health Sciences, Wake Forest School of Medicine, Winston-Salem, NC,; 4 University of Utah, Salt Lake City, UT,; 5 University of Alabama at Birmingham, Birmingham, AL,; 6 University of Illinois, Chicago, IL,; 7 VA Salt Lake City, Salt Lake City, UT,; 8 Houston Veterans Affairs Medical Center and Baylor College of Medicine, Houston, TX,; 9 Tufts Medical Center, Boston, MA,; 10 David Geffen School of Medicine, University of California, Los Angeles, CA, and; 11 VA Salt Lake City Geriatric Research, Education and Clinical Center, Salt Lake City, UT, USA

**Keywords:** aging, chronic kidney disease, clinical epidemiology, renal function decline, pulse wave velocity

## Abstract

Aims: Arterial stiffness increases with both advancing age and chronic kidney disease (CKD) and may contribute to kidney function decline, but evidence is inconsistent. We hypothesized that greater baseline arterial stiffness (assessed as pulse pressure (PP) and carotid-femoral pulse-wave velocity CFPWV)) was independently associated with kidney disease progression over the follow-up period (3.8 years) in the Systolic Blood Pressure Intervention Trial (SPRINT). Materials and methods: 8,815 SPRINT participants were included in the analysis of PP. 592 adults who participated in a SPRINT ancillary study that measured CFPWV were included in subgroup analyses. Cox proportional hazards analysis was used to examine the association between PP and time to kidney disease progression endpoints: (A) incident estimated glomerular filtration rate (eGFR) < 60 mL/min/1.73m^2^ in non-CKD participants at baseline; (B) 50% decline in eGFR, initiation of dialysis, or transplant in those with baseline CKD. Mixed model analyses examined the association of baseline PP/CFPWV with follow-up eGFR. Results and conclusion: Mean ± SD age was 68 ± 10 years, baseline PP was 62 ± 14 mmHg, and CFPWV was 10.8 ± 2.7 m/s. In the fully adjusted model, PP ≥ median was associated with an increased hazard of kidney disease progression endpoints (HR: 1.93 (1.43 – 2.61)). The association remained significant in individuals without (2.05 (1.47 – 2.87)) but not with baseline CKD (1.28 (0.55 – 2.65)). In fully adjusted models, higher baseline PP associated with eGFR decline (p < 0.0001 (all, CKD, non-CKD)), but baseline CFPWV did not. Among older adults at high risk for cardiovascular events, baseline PP was associated with kidney disease progression.

## Introduction 

Arterial stiffness increases both with advancing age [1, 2] and as kidney function declines (chronic kidney disease (CKD)) [3, 4, 5] and is an important independent predictor of incident cardiovascular events and mortality [6, 7, 8, 9]. Aortic stiffness may also contribute to reduced kidney function (lower estimated glomerular filtration rate (eGFR)) by transferring excessive flow pulsatility to a susceptible kidney microvasculature, leading to dynamic constriction and/or vessel loss [10]. 

Evidence whether increased arterial stiffness is associated either cross-sectionally with kidney function [10, 11] or longitudinally with decline in kidney function is inconsistent. Higher baseline arterial stiffness was independently associated with incident CKD (eGFR < 60 mL/min/1.73m^2^) [12, 13] and eGFR decline [14, 15] in several cohorts of community-dwelling adults; however, evidence is not consistent across studies [13, 16] and analyses have included individuals with diabetes mellitus, who likely have greater baseline arterial stiffness [17]. Evidence regarding the association of arterial stiffness with kidney function decline in individuals with prevalent CKD (reduced eGFR) is also inconsistent [18, 19, 20, 21, 22]. 

Accordingly, the purpose of the present study was to test the hypothesis that greater baseline arterial stiffness was independently associated with decline in kidney function over the follow-up period in older adults without baseline diabetes mellitus who participated in the recently completed Systolic Blood Pressure Intervention Trial (SPRINT). We hypothesized that greater baseline arterial stiffness, as measured in the entire cohort by pulse pressure (PP) (a surrogate index of arterial stiffness) [23] and by carotid-femoral pulse-wave velocity (CFPWV) in a subgroup who participated in an ancillary study [24], would be associated with kidney disease progression over the follow-up period. We also explored any differences in these associations in individuals with and without baseline CKD. 

## Materials and methods 

### Study design 

SPRINT was a multi-center, randomized, controlled trial in adults at high risk for cardiovascular events comparing standard (target systolic blood pressure (SBP) of < 140 mmHg) to intensive (target SBP of < 120 mmHg) blood pressure control, with a primary composite endpoint of myocardial infarction, other acute coronary syndromes, stroke, heart failure, or death from cardiovascular causes, as described previously [25, 26]. The protocol for the trial is publically available [27]. Briefly, 9,361 adults ≥ 50 years of age with SBP of 130 – 180 mmHg and increased risk of cardiovascular events (but free from diabetes mellitus and prior stroke) were recruited from 102 clinical sites between November 2010 and March 2013. Detailed inclusion and exclusion criteria have been described previously [25]. 

For the present analysis, participants were classified based on the presence or absence of baseline CKD, defined in SPRINT as a baseline eGFR < 60 mL/min/1.73m^2^ using the four-variable Modification of Diet in Renal Disease (MDRD) Study equation [25]. Of the 9,361 participants with baseline data, 38 were missing information on kidney disease progression endpoints (defined below), and 493 were missing one or more included covariates, leaving a total cohort of 8,815 for analysis of the association of PP with kidney disease endpoints. The most frequently missed covariate was urinary albumin-to-creatinine ratio (ACR) (n = 422). 

652 SPRINT participants from 11 clinical sites enrolled in an ancillary study that measured CFPWV, as described in detail previously [24]. Due to the limited number of kidney disease progression endpoints in this ancillary study (n = 18), the dependent variable for this analysis was instead defined as change in eGFR over the follow-up period. Of the 652 ancillary study participants, 61 were missing one or more covariates, leaving a total cohort of 591 for analysis of the association of CFPWV with change in eGFR. The most frequently missing covariate was urinary ACR (n = 33). 

All participants provided written informed consent. This study was approved by the investigational review boards at the participating centers and was conducted in adherence with the Declaration of Helsinki. 

### Study variables 

**Exposure variables**

PP, a surrogate of arterial stiffness [23], was calculated as SBP – diastolic blood pressure (DBP). There is a significant correlation between PP and CFPWV in the SPRINT CFPWV ancillary study (R = 0.25, p < 0.0001, n = 652) [24]. Blood pressure was measured during the baseline randomization study visit as the mean of three office blood pressure measurements obtained in the seated position using an automated device (Omron Healthcare, Lake Forest, IL, USA) after a 5-minute rest period, as described in detail previously [26, 27, 28]. 

CFPWV was measured using the SphygmoCor CPV system device with software version 9.0 (AtCor Medical, Itasca, IL, USA) following a standard protocol, as described in detail previously [24]. 

**Outcome variables**

In analyses where PP was the predictor, the primary outcome was time to achieve a kidney disease progression endpoint. For participants with baseline eGFR < 60 mL/min/1.73m^2^ (baseline CKD), the kidney disease progression endpoint was a composite of decrease in the eGFR of 50% or more (confirmed by a subsequent laboratory test at least 90 days apart) or the development of end-stage renal disease (ESRD) requiring long-term dialysis or kidney transplantation, as defined previously in the SPRINT study [25]. The non-CKD group included individuals with a baseline eGFR ≥ 60 mL/min/1.73m^2^ (as well as individuals with unknown CKD status at baseline) [25]. The kidney disease progression endpoint in this group was incident CKD, defined as a decrease in eGFR of ≥ 30% to a value of < 60 mL/min/1.73m^2^ [25]. 

In analyses where CFPWV was the predictor, eGFR over time was the outcome variable, using a random intercept, random slope mixed model analysis using all available eGFR measurements. This analysis was also performed as a secondary endpoint with PP as the predictor variable. 

**Covariates and stratification variable**

Baseline characteristics potentially related to arterial stiffness and kidney function decline, all measured at baseline, were selected a priori as covariates for this analysis. Baseline questionnaires and interviews were administered by trained clinical staff. Race and smoking status were determined by self-report. History of cardiovascular disease (CVD) and heart failure were determined by a detailed medical history collected at screening [25, 26]. 

Body-mass index (BMI) was calculated as weight in kilograms divided by height in m^2^. Urinary ACR was calculated as urinary albumin/urinary creatinine (mg/g), using a spot urine. Number of anti-hypertensive agents at baseline (prior to randomization) was determined as described previously [25]. 

### Statistical analyses 

The association of baseline PP and kidney disease progression endpoints was analyzed using Cox proportional hazards analysis. PP was considered as both a continuous variable as well as dichotomized as above and below the median PP. The association between CFPWV (dichotomized by median baseline CFPWV) and change in eGFR over time (interaction of baseline CFPWV × time as a predictor) was analyzed using a mixed model with random intercept and random slopes incorporating all available measurements of eGFR. Natural log-transformed eGFR values were used in these analyses. 

In each analysis, the initial model was unadjusted, with subsequent multivariable models adjusting for age, sex, race, and randomized treatment arm (model 1), model 1 plus CVD, heart failure, smoking, BMI, baseline eGFR (except for mixed model), and urinary ACR (model 2), and model 2 plus number of antihypertensive agents at baseline (model 3). Finally, mean arterial pressure (MAP) and heart rate were added (model 4). Of note, PP and MAP and were weakly correlated, thus unlikely to be collinear. We also evaluated the interaction of PP with sex and race. It was decided a priori to perform stratified analyses according to CKD and non-CKD groups regardless of the interaction term, as kidney disease progression may differ in individuals with and without baseline CKD. 

As a secondary analysis, the association between PP (dichotomized by median baseline PP) and change in eGFR over time (interaction of baseline PP × time as a predictor) was analyzed using a mixed model with random intercept and random slopes incorporating all available measurements of eGFR. Natural log-transformed eGFR values were used in these analyses. 

Indices of kidney function decline and covariates at baseline were summarized above and below the median PP/CFPWV, and are presented as mean (standard deviation) or median (interquartile range) for continuous variables and n (%) for categorical variables. Comparisons between PP/CFPWV groups were made using a χ^2^-test for categorical data and an independent samples t-test for continuous variables. Non-normally distributed variables were log-transformed (urinary ACR) or compared between groups using the Wilcoxon rank sum nonparametric test (eGFR slope). Two-tailed values of p < 0.05 were considered statistically significant for all analyses. All statistical analyses were performed using SAS version 9.4. 

## Results 

### Pulse pressure 

Among 8,815 SPRINT participants with complete data for PP analyses, the mean ± SD age was 68 ± 10 years and 61% (n = 5,049) were White. The mean PP was 62 ± 14 mmHg, and mean baseline eGFR was 72 ± 21 mL/min/1.73m^2^. Individuals with a higher PP were more likely to be older, female, White, have prevalent CVD, heart failure, and CKD, have higher MAP and urinary ACR, have a lower baseline BMI, eGFR and heart rate, use more antihypertensive agents, and less likely to smoke (Table 1). The baseline participant characteristics broken down into subgroups with and without baseline CKD are shown in Online Supplemental Table 1 and 2. There were 243 (2.6%) kidney disease progression endpoints over a median follow-up of 3.8 years. Both the number of kidney disease progression endpoints and the annual decline in eGFR were greater in individuals with a higher PP. 

### Pulse pressure and kidney disease progression endpoints 

**Figure 1.**
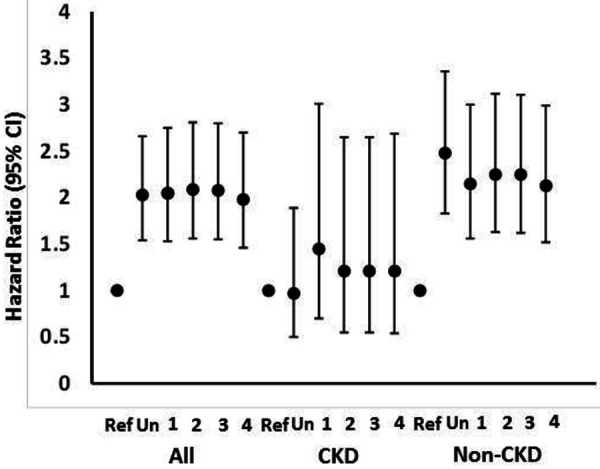
Hazard ratios (95% confidence intervals) for the association of pulse pressure above the median (≥ 60 mmHg vs. Ref (< 60 mmHg)) with kidney disease progression endpoints. Kidney disease progression endpoints are defined as incident chronic kidney disease (CKD) (a decrease in estimated glomerular filtration rate (eGFR) of > 30% to a value of < 60 mL/min/1.73m^2^) in non-CKD participants at baseline and a) 50% decline in eGFR, b) initiation of dialysis, or c) transplant in those participants with CKD at baseline. Models are unadjusted (Un), adjusted for age, sex, race, and randomized treatment arm [1], covariates in model 1 plus cardiovascular disease, heart failure, smoking, body mass index, eGFR, urine albumin-to-creatinine ratio [2]; covariates in model 2 plus number of antihypertensive medications at baseline [3], and covariates in model 3 plus mean arterial pressure and heart rate [4].

In both unadjusted and adjusted analyses, higher PP (above the median; ≥ 60 mmHg) was associated with an increased hazard of a kidney disease progression endpoint compared to the reference group (PP below the median; < 60 mmHg) in all participants (Table 2) (Figure 1). This association was only slightly attenuated in the final model (model 4) adjusted for baseline MAP and heart rate. Results were similar when PP was considered as a continuous variable. The interaction between baseline CKD and PP was significant in model 4 (p = 0.004). The association remained significant in individuals without baseline CKD, but not individuals with baseline CKD; however, the total sample size, as well as number of events (n = 35, 1.4%), were smaller in the latter group. The interaction terms for PP with sex and PP with race were not statistically significant (p ≥ 0.13). 

### Carotid-femoral pulse-wave velocity 

591 SPRINT PWV ancillary study participants were included in the cross-sectional analysis with CFPWV as the predictor variable. Among these participants, the mean ± SD age was 72 ± 10 years, and 67% were White. Individuals with a higher CFPWV were more likely to be older and have a higher MAP (Table 3). The mean CFPWV was 10.8 ± 2.7 m/s. Baseline participant characteristics by CKD status are shown in Online Supplemental Table 3 and 4. There were 18 kidney disease progression endpoints in this ancillary study, too few to evaluate as the dependent variable. 

### Carotid-femoral pulse-wave velocity and decline in estimated glomerular filtration rate 

In fully adjusted analyses (model 4) including all participants, higher CFPWV was not associated with decline in eGFR, incorporating all available time points where eGFR was measured (baseline CFPWV × time interaction p = 0.34). Compared to baseline CFPWV above the median, annual change in lneGFR in individuals with baseline CFPWV below the median was 0.005 (95% confidence interval: –0.005 to 0.015) (model 4). Stratified analyses were performed, as decided a priori, according to CKD and non-CKD groups. The association remained non-significant in individuals with and without baseline CKD. Compared to baseline CFPWV above the median, annual change in lneGFR in individuals with baseline CFPWV below the median in individuals with baseline CKD was 0.0020 (–0.009 to 0.013) (baseline CFPWV × time interaction p = 0.72) (model 4). Compared to baseline CFPWV above the median, annual change in lneGFR in individuals without baseline CFPWV below the median in individuals without baseline CKD was 0.011 (–0.009 to 0.030) (baseline CFPWV × time interaction p = 0.28) (model 4). 

### Relation between pulse pressure and decline in estimated glomerular filtration rate 

As a secondary endpoint, we also considered the association of PP with change in eGFR over time. PP was associated with a greater decline in eGFR, in the entire cohort in the fully adjusted model (model 4; baseline PP × time interaction p < 0.0001). Compared to baseline PP below the median, annual change in lneGFR in individuals with baseline PP above the median was –0.011 (–0.014 to –0.009). Again, stratified analyses were performed, as decided a priori, according to CKD and non-CKD groups. In individuals with baseline CKD, compared to baseline PP below the median, annual change in lneGFR in individuals with baseline PP above the median was –0.014 (–0.020 to –0.009) (baseline PP × time interaction p < 0.0001) (model 4). In individuals without baseline CKD, compared to baseline PP below the median, annual change in lneGFR in individuals with baseline PP above the median was –0.010 (–0.013 to –0.008) (baseline PP × time interaction p < 0.0001) (model 4). 

## Discussion 

In older individuals with hypertension and at high risk for cardiovascular events, higher arterial stiffness, as measured by PP, was associated with an increased hazard of a kidney disease progression over a median follow-up of 3.8 years. This association was significant in individuals without baseline CKD, but not in those with baseline CKD. Of note, the sample size and number of events was smaller for the CKD group. However, both individuals with and without baseline CKD demonstrated an association of higher baseline PP with decline in eGFR over time. In contrast, we failed to demonstrate an association using the gold-standard measurement of CFPWV as the index of arterial stiffness; notably, the sample size was much smaller in this ancillary study. 

Evidence to date regarding the association of arterial stiffness with kidney function and subsequent decline in kidney function has been inconsistent. In cohorts of community-based adults, arterial stiffness (measured by CFPWV or PP), has been both independently associated [10] and not associated with eGFR cross-sectionally [11, 16]. Longitudinally, greater baseline CFPWV has predicted eGFR decline or incident CKD in some, but not other populations of community-based adults [12, 13, 14, 16]. Previous analyses with PP as the predictor variable have been similarly inconsistent [12, 13, 14, 16]. Notably, these previous cohorts have all included individuals with diabetes mellitus, while SPRINT participants were free from diabetes at baseline. In relatively small studies of individuals with prevalent CKD, greater baseline CFPWV has been associated with kidney disease progression in some [20, 29], but not other [18] cohorts. Recently, baseline CFPWV was associated with kidney disease progression in a large number of participants (n = 2,795) with prevalent CKD in the Chronic Renal Insufficiency Cohort [22]. Several previous analyses have also found no independent association between baseline PP and decline in kidney function in individuals with prevalent CKD [18, 20]. 

In participants in the SPRINT study, we found an independent association of greater baseline PP with kidney disease progression endpoints, defined as incident CKD (a decrease in eGFR of > 30% to a value of < 60 mL/min/1.73m^2^) in non-CKD participants at baseline, and either a 50% decline in eGFR, initiation of dialysis, or transplant in participants with CKD at baseline. There was a significant interaction term between PP and baseline CKD status in this analysis, such that an association remained for individuals without but not with baseline CKD. However, when change in eGFR over time was considered as a secondary endpoint, the association of PP with this endpoint was significant in both individuals with and without baseline CKD. 

Mechanistically, with stiffening of the large elastic arteries, the microvasculature is exposed to highly pulsatile pressure and flow, promoting microvascular damage [30]. Excessive pulsatile pressure in glomerular capillaries can promote reductions in kidney function, as the kidney is a high-flow, low-impedance organ that is particularly susceptible to pulsatile damage [31]. Both dynamic constriction and vessel loss may contribute to reductions in eGFR [10]. This is supported by a mediation analysis from the Age, Gene/Environment Susceptibility-Reykjavik Study (AGES), which demonstrated that the cross-sectional association of higher CFPWV with lower eGFR was mediated in part by increased pulsatility index, lower arterial volume in the cortex, and higher kidney vascular resistance, suggesting an increase transmission of pulsatile energy to the kidneys [10]. 

Notable strengths of this study include a large sample size available for the PP analyses and including endpoints and a large number of important covariates in the setting of a clinical trial. Additionally, we separately considered progression in individuals with and without baseline CKD, as well as two predictor variables representing arterial stiffness. Our findings are notable as they represent the largest study to date examining the association of markers of arterial stiffness with kidney function decline in a population both with and without baseline CKD. 

There are also important limitations of this analysis. The results are associative, and residual confounding may exist, including variables that were not assessed, such as level of inflammation. There were not enough kidney disease events to consider this endpoint in the analyses with CFPWV as the predictor variable because of smaller sample size. The SPRINT cohort did not include younger adults with less CVD burden, nor did it include individuals with stroke or prevalent diabetes; thus, these results may not apply to these populations. Overall, participants who were included in SPRINT may not resemble the broader population of older adults with or without CKD, thus limiting the external validity of the results. Additionally, we were not able to consider individual classes of antihypertensive medications as covariates. 

In conclusion, among adults at high risk for cardiovascular events without history of diabetes or stroke, PP was associated with kidney disease progression endpoints as well as change in eGFR over time. The latter association remained significant in those with and without baseline CKD, while the former association was observed in the baseline non-CKD subgroup. In contrast, CFPWV was not associated with decline in eGFR; however, power was limited for this analysis. Overall, these results are consistent with the hypothesis that arterial stiffness may contribute to kidney disease progression. 

## Acknowledgment 

The SPRINT investigators acknowledge the contribution of study medications (azilsartan and azilsartan combined with chlorthalidone) from Takeda Pharmaceuticals International, Inc. All components of the SPRINT study protocol were designed and implemented by the investigators. The investigative team collected, analyzed, and interpreted the data. All aspects of manuscript writing and revision were carried out by the coauthors. The content is solely the responsibility of the authors and does not necessarily represent the official views of the NIH, the U.S. Department of Veterans Affairs, or the United States Government. For a full list of contributors to SPRINT, please see the supplementary acknowledgement list: https://www.sprinttrial.org/public/dspScience.cfm.

## Funding 

The Systolic Blood Pressure Intervention Trial is funded with Federal funds from the National Institutes of Health (NIH), including the National Heart, Lung, and Blood Institute (NHLBI), the National Institute of Diabetes and Digestive and Kidney Diseases (NIDDK), the National Institute on Aging (NIA), and the National Institute of Neurological Disorders and Stroke (NINDS), under Contract Numbers HHSN268200900040C, HHSN268200900046C, HHSN2​6​8​2009​000​47C, HHSN268200900048C, HHSN2​6​8​2​0​0900049C, and Inter-Agency Agreement Number A-HL-13-002-001. It was also supported in part with resources and use of facilities through the Department of Veterans Affairs. 

We also acknowledge the support from the following CTSAs funded by NCATS: CWRU: UL1TR000439, OSU: UL1RR025755, U Penn: UL1RR024134 & UL1TR000003, Boston: UL1RR025771, Stanford: UL1TR000093, Tufts: UL1RR025752, UL1TR000073 & UL1TR001064, University of Illinois: UL1TR000050, University of Pittsburgh: UL1TR000005, UT Southwestern: 9U54TR000017-06, University of Utah: UL1TR000105-05, Vanderbilt University: UL1TR000445, George Washington University: UL1TR000075, University of CA, Davis: UL1TR000002, University of Florida: UL1TR000064, University of Michigan: UL1TR000433, Tulane University: P30GM103337 COBRE Award NIGMS, Wake Forest University: UL1TR001420. 

The PWV ancillary study was supported by NHLBI (Mark Supiano: R01HL107241). Kristen Nowak is supported by NIDDK (K01DK103678). Anna Jovanovich is supported by Veterans Administration CDA 5IK2CX001030-03. 

## Conflict of interest 

DEW has participated in advisory boards for Janssen and Akebia and has represented Dialysis Clinic Inc. in advisory boards for Keryx and Relypsa. AJ receives study drug from Shire. 

**Table 1.** Baseline characteristics of study participants in the entire cohort by baseline pulse pressure.

Variable	Baseline PP below the median (< 60 mmHg) (n = 4,249)	Baseline PP above the median (≥ 60 mmHg) (n = 4,566)	p-value
Age, y	64 ± 8	72 ± 9	< 0.0001
Sex, n (%) male	2,940 (69%)	2,765 (61%)	< 0.0001
Race, n (%) white	2,245 (53%)	2,804 (61%)	< 0.0001
Study randomization, n (%) intensive treatment	2,121 (50%)	2,300 (50%)	0.67
Prevalent CVD, n (%)	751 (18%)	1,040 (23%)	< 0.0001
Prevalent heart failure, n (%)	129 (3%)	189 (4%)	0.006
Prevalent CKD, n (%)	1,050 (25%)	1,481 (32%)	< 0.0001
Smoking status, n (%)			< 0.0001
Never smoked	1,848 (43%)	2,027 (44%)	
Former smoker	1,675 (39%)	2,084 (46%)	
Current smoker	730 (17%)	455 (10%)	
MAP, mmHg	98.0 ± 10.9	99.3 ± 11.9	< 0.0001
Body mass index, kg/m^2^	30.8 ± 5.9	29.0 ± 5.5	< 0.0001
eGFR, mL/min/1.73m^2^	74 ± 21	70 ± 21	< 0.0001
Urinary albumin to creatinine ratio	7.9 (5.0, 16.6)	11.4 (6.5, 26.9)	< 0.0001
Heart rate, beats per minute	71 ± 12	66 ± 11	< 0.001
Antihypertensive agents, no./patient			< 0.0001
0	431 (10%)	434 (9%)	
1	1,503 (33%)	1,426 (30%)	
2	1,545 (34%)	1,631 (34%)	
3	793 (18%)	1,061 (22%)	
4	222 (5%)	261 (5%)	
Pulse pressure, mmHg	50 ± 7	73 ± 11	< 0.0001
Kidney disease progression endpoints, n (%)	75 (1.8%)	159 (3.5%)	< 0.0001
eGFR slope, mL/min/1.73m^2^ per year	–0.3 (–2.2, 1.6)	–0.8 (–3.1, 1.1)	< 0.0001

Data are mean ± SD, median (IQR), or n (%). PP = pulse pressure; CVD = cardiovascular disease; CKD = chronic kidney disease; eGFR = estimated glomerular filtration rate (Modification of Diet in Renal Disease equation); MAP = mean arterial pressure. Kidney disease progression endpoints are defined as incident CKD (a decrease in eGFR of > 30% to a value of < 60 mL/min/1.73m^2^) in non-CKD participants at baseline and a) 50% decline in eGFR, b) initiation of dialysis, or c) transplant in those participants with CKD at baseline.

**Table 2.** Associations (hazard ratio (95% CI)) of baseline arterial stiffness (pulse pressure) with kidney disease progression.

All participants	Baseline PP below the median (< 60 mmHg) (n = 4,249)	Baseline PP above the median (≥ 60 mmHg) (n = 4,566)	Continuous (per mmHg higher baseline PP) (n = 8,815)
Unadjusted	Ref	2.03 (1.54, 2.67)	1.03 (1.02, 1.04)
Model 1	Ref	2.05 (1.53, 2.75)	1.03 (1.02, 1.04)
Model 2	Ref	2.09 (1.56, 2.81)	1.03 (1.02, 1.04)
Model 3	Ref	2.08 (1.55, 2.80)	1.03 (1.02, 1.04)
Model 4	Ref	1.93 (1.43, 2.61)	1.03 (1.02, 1.04)
Prevalent CKD	Baseline PP below the median (< 63 mmHg) (n = 1,050)	Baseline PP above the median (≥ 63 mmHg) (n = 1,481)	Continuous (per mmHg higher baseline PP) (n = 2,531)
Unadjusted	Ref	0.97 (0.50, 1.89)	1.00 (0.98, 1.03)
Model 1	Ref	1.45 (0.70, 3.01)	1.02 (1.00, 1.04)
Model 2	Ref	1.21 (0.55, 2.65)	1.01 (0.99, 1.04)
Model 3	Ref	1.21 (0.55, 2.65)	1.01 (0.99, 1.04)
Model 4	Ref	1.28 (0.55, 2.65)	1.01 (0.99, 1.04)
Non-CKD	Baseline PP below the median (< 59 mmHg) (n = 3,199)	Baseline PP above the median (≥ 59 mmHg) (n = 3,085)	Continuous (per mmHg higher baseline in PP) (n = 6,284)
Unadjusted	Ref	2.48 (1.83, 3.35)	1.04 (1.03, 1.05)
Model 1	Ref	2.15 (1.56, 2.97)	1.04 (1.03, 1.05)
Model 2	Ref	2.25 (1.62, 3.11)	1.04 (1.03, 1.05)
Model 3	Ref	2.24 (1.62, 3.11)	1.04 (1.03, 1.05)
Model 4	Ref	2.05 (1.47, 2.87)	1.04 (1.03, 1.05)

PP = pulse-pressure; CKD = chronic kidney disease; CVD = cardiovascular disease; eGFR = estimated glomerular filtration rate; ACR = albumin to creatinine ratio; MAP = mean arterial pressure. Kidney disease progression endpoints are defined as incident CKD (a decrease in eGFR of > 30% to a value of < 60 mL/min/1.73m^2^) in non-CKD participants at baseline and a) 50% decline in eGFR, b) initiation of dialysis, or c) transplant in those participants with CKD at baseline. Model 1: adjusted for age, sex, race, and randomized treatment arm; Model 2: adjusted for covariates in model 1 plus CVD, heart failure, smoking, body mass index, eGFR, urine ACR; Model 3: adjusted for covariates in model 2 plus number of antihypertensive medications at baseline; Model 4: adjusted for covariates in model 3 plus MAP and heart rate.

**Table 3.** Baseline characteristics of study participants in the pulse-wave velocity ancillary study by baseline aortic pulse-wave velocity.

Variable	Baseline CFPWV below the median (< 10.6 m/sec) (n = 276)	Baseline CFPWV above the median (≥ 10.6 m/sec) (n = 315)	p-value
Age, y	70 ± 9	74 ± 9	< 0.0001
Sex, n (%) male	192 (61%)	1657 (60%)	0.77
Race, n (%) white	191 (69%)	204 (65%)	0.25
Study randomization, n (%) intensive treatment	137 (50%)	158 (50%)	0.90
Prevalent CVD, n (%)	32 (12%)	48 (15%)	0.20
Prevalent CHF, n (%)	5 (2%)	6 (2%)	0.93
Prevalent CKD, n (%)	106 (34%)	105 (38%)	0.27
Smoking status, n (%)			0.26
Never smoked	131 (48%)	141 (45%)	
Former smoker	130 (47%)	146 (46%)	
Current smoker	15 (5%)	28 (9%)	
MAP, mm Hg	95.8 ± 11.5	98.3 ± 11.3	0.008
Body mass index, kg/m^2^	28.3 ± 5.1	27.7 ± 5.1	0.21
eGFR, mL/min/1.73m^2^	69 ± 21	66 ± 20	0.23
Urinary albumin to creatinine ratio	9.9 (6.0, 25.2)	12.3 (7.1, 28.3)	0.05
Heart rate, beats per minute	66 ± 12	67 ± 20	0.83
Antihypertensive agents, no./patient			0.19
0	20 (7%)	28 (10%)	
1	111 (39%)	104 (36%)	
2	84 (30%)	99 (34%)	
3	45 (16%)	40 (14%)	
4	24 (9%)	21 (7%)	
CFPWV, m/s	8.8 ± 1.3	13.1 ± 2.0	< 0.0001
Pulse pressure, mm Hg	63 ± 14	69 ±15	< 0.0001
eGFR slope, mL/min/1.73m^2 ^per year	–0.3 (–2.6, 1.3)	–0.5 (–2.6, 1.4)	0.82

Data are mean ± SD, median (IQR), or n (%). CFPWV = carotid-femoral pulse-wave velocity; CVD = cardiovascular disease; CKD = chronic kidney disease; eGFR= estimated glomerular filtration rate (Modification of Diet in Renal Disease equation); MAP = mean arterial pressure.

## Supplemental material

Supplemental material. Supplemental Tables
