# Supplementary material for: Arterial stiffness and kidney disease progression in the systolic blood pressure intervention trial
Source: Clin Nephrol. 2020 May 25;94(1):26–35. doi: 10.5414/CN109982 (PMC7814777; doi:10.5414/CN109982)
Supplement: Supplemental material. [file clinnephrol-94-026-S01.pdf]

Supplemental Material

Arterial Stiffness and Kidney Function Decline in SPRINT

Kristen L Nowak, Michel Chonchol, Anna Jovanovich, Zhiying You, Walter T Ambrosius, Monique E Cho, Stephen Glasser, James Lash, Debra L Simmons, Addison Taylor, Daniel Weiner, Anjay Rastogi, Suzanne Oparil, Mark A. Supiano

Supplemental Table 1. Baseline Characteristics of Study Participants from the Entire Cohort with Baseline CKD by Baseline Pulse Pressure

Variable	Baseline PP Below the Median (< 63 mmHg) (n=1050)	Baseline PP Above the Median (≥ 63 mmHg) (n=1481)	*P-Value*
**Age**, y	67 ± 9	75 ± 8	<0.0001
**Sex**, n (%) Male	671 (64%)	864 (58%)	0.005
**Race**, n (%) White	649 (62%)	1044 (71%)	<0.0001
**Study Randomization**, n (%) intensive treatment	534 (51%)	742 (50%)	0.71
**Prevalent CVD**, n (%)	212 (20%)	413 (28%)	<0.0001
**Prevalent Heart Failure**, n (%)	60 (6%)	100 (7%)	0.29
**Smoking Status**, n (%)			0.001
Never smoked	485 (46%)	661 (45%)	
Former smoker	452 (43%)	717 (48%)	
Current smoker	113 (11%)	103 (7%)	
**MAP**, mm Hg	95.7 ± 11.1	96.8 ± 11.7	0.02
**Body mass index**, kg/m^2	30.6 ± 6.1	28.7 ± 5.4	<0.0001
**eGFR**, ml/min/1.73m^2	48 ± 10	48 ± 9	0.21
**Urinary albumin to creatinine ratio**	10.6 (5.2, 36.0)	15.5 (7.4, 48.6)	<0.0001
**Heart rate**, beats per minute	70 ± 12	65 ± 11	<0.0001

**Variable**	**Baseline PP Below the Median** (< 63 mmHg) (n=1050)	**Baseline PP Above the Median** (≥ 63 mmHg) (n=1481)	***P-Value***
**Antihypertensive agents**, no./patient			0.02
0	54 (5%)	61 (4%)	
1	298 (28%)	347 (23%)	
2	364 (35%)	538 (36%)	
3	258 (25%)	429 (29%)	
4	76 (7%)	106 (7%)	
**Pulse Pressure**, mm Hg	50 ± 7	74 ± 12	<0.0001
**Kidney disease progression endpoints**, n (%)	15 (1.4%)	20 (1.4%)	0.87
**eGFR slope**, ml/min/1.73m^2 per year	0.0 (-1.5, 1.7)	-0.5 (-2.4, 1.1)	<0.0001

Data are mean±SD. median (IQR), or n (%). PP, pulse pressure; CVD, cardiovascular disease; CKD, chronic kidney disease; eGFR, estimated glomerular filtration rate (Modification of Diet in Renal Disease equation); mean arterial pressure, MAP. Baseline CKD was defined in SPRINT as a baseline estimated glomerular filtration rate (eGFR) <60 ml/min/1.73 m^2 using the four-variable Modification of Diet in Renal Diseases (MDRD) Study equation. Kidney disease progression endpoints are defined as a) 50% decline in eGFR, b) initiation of dialysis, or c) transplant in those participants with CKD at baseline.

Supplemental Table 2. Baseline Characteristics of Study Participants from the Entire Cohort without Baseline CKD by Baseline Pulse Pressure

Variable	**Baseline PP Below the Median** (<59 mmHg) (n=3199)	**Baseline PP Above the Median** (≥ 59 mmHg) (n=3085)	***P-Value***
**Age**, y	63 ± 8	70 ± 9	<0.0001
**Sex**, n (%) Male	2269 (71%)	1901 (62%)	<0.0001
**Race**, n (%) White	1596 (50%)	1760 (57%)	<0.0001
**Study Randomization**, n (%) intensive treatment	1587 (50%)	1558 (51%)	0.48
**Prevalent CVD**, n (%)	539 (17%)	627 (20%)	0.0004
**Prevalent Heart Failure**, n (%)	69 (2%)	89 (3%)	0.07
**Smoking Status**, n (%)			<0.0001
Never smoked	1359 (43%)	1366 (44%)	
Former smoker	1223 (38%)	1367 (44%)	
Current smoker	617 (19%)	352 (11%)	
**MAP**, mm Hg	98.8 ± 10.7	100.5 ± 11.8	<0.0001
**Body mass index**, kg/m^2	30.9 ± 5.8	29.2 ± 5.6	<0.0001
**eGFR**, ml/min/1.73m^2	82 ± 16	81 ± 16	0.0003
**Urinary albumin to creatinine ratio**	7.5 (5.0, 14.3)	10.1 (6.3, 20.0)	<0.0001
**Heart rate**, beats per minute	71 ± 12	66 ± 12	<0.0001

**Variable**	**Baseline PP Below the Median** (<59 mmHg) (n=3206)	**Baseline PP Above the Median** (≥ 59 mmHg) (n=3090)	***P-Value***
**Antihypertensive agents**, no./patient			0.0008
0	348 (11%)	341 (11%)	
1	1126 (35%)	1005 (33%)	
2	1103 (35%)	1007 (33%)	
3	490 (15%)	591 (19%)	
4	132 (4%)	141 (5%)	
**Pulse Pressure**, mm Hg	50 ± 7	72 ± 10	<0.0001
**Kidney disease progression endpoints**, n (%)	60 (1.9%)	139 (4.5%)	<0.0001
**eGFR slope**, ml/min/1.73m^2 per year	-0.4 (-2.5, 1.6)	-1.0 (-3.6, 1.1)	<0.0001

Data are mean±SD., median (IQR), or n (%). PP, pulse pressure; CVD, cardiovascular disease; CKD, chronic kidney disease; eGFR, estimated glomerular filtration rate (Modification of Diet in Renal Disease equation); mean arterial pressure, MAP. The non-CKD group included individuals with a baseline eGFR ≥60 ml/min/1.73 m^2 (as well as individuals with unknown CKD status at baseline). Kidney disease progression endpoints are defined as incident CKD (a decrease in eGFR of >30% to a value of <60 ml/min/1.73 m^2).

Supplemental Table 3. Baseline Characteristics of Study Participants with Baseline CKD in the Pulse-Wave Velocity Ancillary Study by Baseline Carotid-Femoral Pulse-Wave Velocity

**Variable**	**Baseline CFPWV Below the Median** (< 10.7 m/sec) (n=106)	**Baseline CFPWV Above the Median** (≥ 10.7 m/sec) (n=105)	***P-Value***
**Age**, y	69 ± 10	75 ± 9	<0.0001
**Sex**, n (%) Male	59 (56%)	67 (64%)	0.23
**Race**, n (%) White	63 (59%)	72 (69%)	0.17
**Study Randomization**, n (%) intensive treatment	57 (54%)	51 (49%)	0.45
**Prevalent CVD**, n (%)	20 (20%)	19 (18%)	0.75
**Prevalent Heart Failure**, n (%)	3 (3%)	2 (2%)	1.00
**Smoking Status**, n (%)			0.30
Never smoked	50 (47%)	54 (51%)	
Former smoker	45 (43%)	46 (44%)	
Current smoker	11 10%)	5 (5%)	
**MAP**, mm Hg	94.9 ± 12.1	97.9 ± 11.7	0.06
**Body mass index**, kg/m^2	28.3 ± 5.4	27.2 ± 4.5	0.13
**eGFR**, ml/min/1.73m^2	46 ± 11	46 ± 11	0.97
**Urinary albumin to creatinine ratio**	14.4 (6.7, 55.4)	13.7 (8.0, 51.5)	0.90
**Heart rate**, beats per minute	64 ± 11	67 ± 11	0.08

Variable	**Baseline CFPWV Below the Median** (< 10.7 m/sec) (n=106)	**Baseline CFPWV Above the Median** (≥ 10.7 m/sec) (n=105)	***P-Value***
**Antihypertensive agents**, no./patient			0.03
0	2 (2%)	9 (9%)	
1	29 (27%)	34 (32%)	
2	32 (30%)	38 (36%)	
3	26 (28%)	15 (14%)	
4	154 (14%)	9 (9%)	
**CFPWV**, m/s	8.7 ± 1.4	13.0 ± 1.8	<0.0001
**Pulse Pressure**, mm Hg	62 ± 14	68 ±15	0.003
**eGFR slope**, ml/min/1.73m^2 per year	0.0 (-1.8, 1.4)	-0.2 (-1.7, 1.2)	0.29

Data are mean±SD., median (IQR), or n (%). CFPWV, carotid-femoral pulse-wave velocity; CVD, cardiovascular disease; CKD, chronic kidney disease; eGFR, estimated glomerular filtration rate (Modification of Diet in Renal Disease equation); MAP, mean arterial pressure. Baseline CKD was defined in SPRINT as a baseline estimated glomerular filtration rate (eGFR) <60 ml/min/1.73 m^2 using the four-variable Modification of Diet in Renal Diseases (MDRD) Study equation.

Supplemental Table 4. Baseline Characteristics of Study Participants without Baseline CKD in the Pulse-Wave Velocity Ancillary Study by Baseline Carotid-Femoral Pulse-Wave Velocity

Variable	**Baseline CFPWV Below the Median** (< 10.5 m/sec) (n=171)	**Baseline CFPWV Above the Median** (≥ 10.5m/sec) (n=209)	***P-Value***
**Age**, y	70 ± 9	74 ± 9	<0.0001
**Sex**, n (%) Male	133 (64%)	98 (58%)	0.21
**Race**, n (%) White	141 (68%)	119 (70%)	0.66
**Study Randomization**, n (%) intensive treatment	101 (48%)	86 (50%)	0.70
**Prevalent CVD**, n (%)	27 (13%)	13 (8%)	0.09
**Prevalent Heart Failure**, n (%)	3 (1%)	3 (2%)	1.00
**Smoking Status**, n (%)			0.69
Never smoked	91 (44%)	77 (45%)	
Former smoker	101 (48%)	84 (49%)	
Current smoker	17 (8%)	10 (6%)	
**MAP**, mm Hg	96.2 ± 11.1	98.5 ± 11.0	0.05
**Body mass index**, kg/m^2	28.3± 4.9	28.1 ± 5.4	0.70
**eGFR**, ml/min/1.73m^2	80.± 15	79 ± 13	0.50
**Urinary albumin to creatinine ratio**	9.3 (6.0, 20.0)	11.6 (6.3, 23.8)	0.03
**Heart rate**, beats per minute	67 ± 11	67 ± 11	0.34

**Variable**	**Baseline CFPWV Below the Median** (< 10.5 m/sec) (n=171)	**Baseline CFPWV Above the Median** (≥ 10.5 m/sec) (n=209)	***P-Value***
**Antihypertensive agents**, no./patient			0.28
0	20 (10%)	18 (11%)	
1	97 (46%)	61 (36%)	
2	58 (28%)	60 (35%)	
3	22 (11%)	23 (14%)	
4	12 (6%)	9 (5%)	
**CFPWV**, m/s	8.8 ± 1.3	13.1± 2.1	<0.0001
**Pulse Pressure**, mm Hg	63 ± 14	69 ± 14	<0.0001
**eGFR slope**, ml/min/1.73m^2 per year	-0.5 (-3.0, 1.3)	-0.5 (-2.9, 1.4)	0.77

Data are mean±SD., median (IQR), or n (%). CFPWV, carotid-femoral pulse-wave velocity; CVD, cardiovascular disease; CKD, chronic kidney disease; eGFR, estimated glomerular filtration rate (Modification of Diet in Renal Disease equation); MAP, mean arterial pressure. The non-CKD group included individuals with a baseline eGFR ≥60 ml/min/1.73 m^2 (as well as individuals with unknown CKD status at baseline).
